# Obesity and PCOS radically alters the snRNA composition of follicular fluid extracellular vesicles

**DOI:** 10.3389/fendo.2023.1205385

**Published:** 2023-06-19

**Authors:** Brandon A. Wyse, Reza Salehi, Stewart J. Russell, Mugundhine Sangaralingam, Sahar Jahangiri, Benjamin K. Tsang, Clifford L. Librach

**Affiliations:** ^1^ Research Department, CReATe Fertility Centre, Toronto, ON, Canada; ^2^ Chronic Disease Program, Ottawa Hospital Research Institute, Ottawa, ON, Canada; ^3^ Departments of Obstetrics and Gynecology & Cellular and Molecular Medicine, University of Ottawa, Ottawa, ON, Canada; ^4^ CReATe Biobank, Toronto, ON, Canada; ^5^ Department of Obstetrics and Gynecology, University of Toronto, Toronto, ON, Canada; ^6^ Department of Physiology, University of Toronto, Toronto, ON, Canada; ^7^ Biological Sciences, DAN Women & Babies Research Program, Sunnybrook Research Institute, Toronto, ON, Canada

**Keywords:** PCOS (polycystic ovarian syndrome), obesity, smallRNA, miRNA, smallRNASeq, extracellular vesicles (EVs), exosome (vesicle)

## Abstract

**Introduction:**

The ovarian follicle consists of the oocyte, somatic cells, and follicular fluid (FF). Proper signalling between these compartments is required for optimal folliculogenesis. The association between polycystic ovarian syndrome (PCOS) and extracellular vesicular small non-coding RNAs (snRNAs) signatures in follicular fluid (FF) and how this relates to adiposity is unknown. The purpose of this study was to determine whether FF extracellular vesicle (FFEV)-derived snRNAs are differentially expressed (DE) between PCOS and non-PCOS subjects; and if these differences are vesicle-specific and/or adiposity-dependent.

**Methods:**

FF and granulosa cells (GC) were collected from 35 patients matched by demographic and stimulation parameters. FFEVs were isolated and snRNA libraries were constructed, sequenced, and analyzed.

**Results:**

miRNAs were the most abundant biotype present, with specific enrichment in exosomes (EX), whereas in GCs long non-coding RNAs were the most abundant biotype. In obese PCOS vs. lean PCOS, pathway analysis revealed target genes involved in cell survival and apoptosis, leukocyte differentiation and migration, JAK/STAT, and MAPK signalling. In obese PCOS FFEVs were selectively enriched (FFEVs vs. GCs) for miRNAs targeting p53 signalling, cell survival and apoptosis, FOXO, Hippo, TNF, and MAPK signalling.

**Discussion:**

We provide comprehensive profiling of snRNAs in FFEVs and GCs of PCOS and non-PCOS patients, highlighting the effect of adiposity on these findings. We hypothesize that the selective packaging and release of miRNAs specifically targeting anti-apoptotic genes into the FF may be an attempt by the follicle to reduce the apoptotic pressure of the GCs and stave off premature apoptosis of the follicle observed in PCOS.

## 1 Introduction

Polycystic ovarian syndrome (PCOS) is a common infertility disorder that is estimated to affect 5-10% of women of reproductive age (1). PCOS is a complex, multifactorial and heterogeneous disorder, characterized by two or more of the following: amenorrhea/oligomenorrhea, hirsutism, hyperandrogenism, and polycystic ovaries and is commonly associated with obesity, insulin resistance, and infertility (2–4). Since PCOS is a syndrome, no single diagnostic criterion (such as hyperandrogenism or PCO) is sufficient for clinical diagnosis. PCOS is also associated with a multitude of metabolic abnormalities including glucose intolerance, hyperinsulinemia, dyslipidemia, and diabetes mellitus type II (T2DM) (5, 6). The ovaries of women with PCOS exhibit a varying degree of ovarian follicle growth arrest at the early antral stage, chronic anovulation, and altered granulosa cell proliferation (7, 8). It is difficult to discern the underlying cause due to its complex pathophysiology; however, it appears to be associated with both genetic abnormalities and environmental exposures (9).

Communication between the somatic cells and oocyte is critical to proper follicular development and oocyte maturation; disturbances in this delicately regulated microenvironment may lead to ovarian pathologies, such as PCOS (10–12). This intercellular communication is known to be facilitated, in part, by extracellular vesicles (EVs) which are spherical bodies composed of a lipid bilayer (13). They are subdivided into 3 main categories based on size, membrane components, and biogenesis pathway. Exosomes (EX) range from ~20-120 nm, characterized by the inclusion of tetraspanins in the membrane (CD9, CD63, and/or CD81), and are generated and released by the endosome system. Microparticles (MP) range from ~120 nm-700 nm, contain a similar composition of plasma membrane factors, and are formed by plasma membrane budding. Apoptotic bodies (AB) are up to 5000 nm, contain annexin V, organelles and nuclear components, and are formed during apoptosis and released by plasma membrane blebbing (14–16). Recently, there has been increased interest in follicular signalling and communication *via* EVs in PCOS patients through miRNA and protein, however to date there has not been a whole snRNA profile of EVs in PCOS and no studies have investigated the impact of obesity on these results (17, 18).

One functional biomolecule class transported by EVs are small, non-coding RNAs (snRNAs) which include a wide variety of unique biotypes, including: miRNA, piRNA, snoRNA, rRNA, and tRNA. SnRNAs have the capability to modulate the target cell transcriptional activity and, in the case of the piRNA, alter DNA and histone methylation (19–21). Recently, several studies have investigated the miRNA profile in PCOS patients’ follicular fluid and/or follicular cells (22–26), however very few studies have investigated the complete snRNA repertoire of PCOS follicular fluid (27) and, to our knowledge, no studies have assessed both the follicular fluid (FF) and granulosa cells (GC) from the same follicle using next-generation sequencing.

Obesity is a common comorbidity associated with PCOS (2, 3) and affects all organ systems (28, 29). However, to date, there have been limited reports pertaining to the influence of adiposity on the follicular snRNA profile, independent of PCOS. One study found an interaction between obesity and PCOS contributing to the overall observed miRNA profile (30). Adipose tissue is the largest source of circulating miRNAs, which are now considered a new class of adipokine (31). Therefore, assessing not only the effects of PCOS on the follicular snRNA profile, but also those of adiposity is critical to further understanding the pathogenesis of these diseases.

The aims of this study were to 1) develop a novel sequencing method for the detection of the snRNAome in multiple components of the follicular fluid microenvironment: microparticles (MP), exosomes (EX), EV-depleted follicular fluid (FFD), and granulosa cells (GC) from a single follicle; 2) compare the snRNA profile of MP, EX, FFD, and GC from PCOS matched to non-PCOS patients; 3) bioinformatically elucidate the impact of adiposity on the snRNA profiles in both PCOS and matched non-PCOS patients; 4) identify specifically EV packaged snRNAs and their potential impact on folliculogenesis.

## 2 Materials and methods

### 2.1 Ethics declaration

All subjects provided written informed consent for the donation of their biological waste material, which included the collection of follicular fluid and granulosa cells and extraction of associated de-identified clinical information including age, fertility diagnosis, body mass index, and treatment regime (University of Toronto Research Ethics Board Approval #29236). The request and use of samples for this study was approved by the University of Toronto Research Ethics Board (Approval #29237).

### 2.2 Patient recruitment, stimulation, and sample collection

FF samples and corresponding GC from a single mature antral follicle were collected and cryopreserved by the CReATe Biobank (Toronto, Ontario, Canada; www.createresearchprogram.com/create-biobank/biological-materials) from 35 patients undergoing IVF-ICSI cycles at CReATe Fertility Centre (Toronto, Ontario, Canada) between March 2017 and May 2018. Samples were stored in liquid nitrogen until requested by researchers. FF and GC from a total of 35 patients were requested from the Biobank: 10 Obese non-PCOS, 10 Lean non-PCOS, 10 Lean PCOS, and 5 Obese PCOS. Obesity was defined as a BMI>30 and PCOS was diagnosed using the Rotterdam criteria (4). Patients receiving metformin and patients with endometriosis confirmed by laparoscopy were excluded from this study. Patients were treated using a standard GnRH antagonist protocol, with initial gonadotropin dosing and subsequent adjustments at the discretion of the treating physician. Patients were matched by demographic and stimulation parameters to limit inter-patient variability (**Table S2**).

### 2.3 Determining the minimum volume of FF for EV isolation and snRNAseq

To determine if it was possible to obtain sufficient material from one FF aspirate, a preliminary experiment using FF collected from 18 patients were retrieved from the Biobank. Three pools of FF were aliquoted into 4, 2, 1, 0.5, and 0.25 mL fractions and EVs were isolated using differential centrifugation and the ExoQuick (System Biosciences, CA, USA), as described in detail below. Particles from each fraction were quantified using the NanoSight LM10 (Malvern Analytical, Malvern, UK) to determine both concentration and size. Total protein was isolated from each fraction and quantified using the Qubit Protein Assay (ThermoFisher, ON, Canada), according to the manufacturer’s instructions. RNA was isolated using the Total Exosome RNA and Protein Isolation Kit (ThermoFisher), according to the manufacturer’s instructions. Finally, sequencing libraries were generated using the Small RNA Kit (Norgen Biotek, ON, Canada) and sequenced using a High Output (75 cycle) flow cell on a NextSeq 550 sequencer (1x75bp). Pearson correlations were conducted to determine the impact FF input had on snRNA detection.

### 2.4 Extracellular vesicle isolation

**Figure 1:**
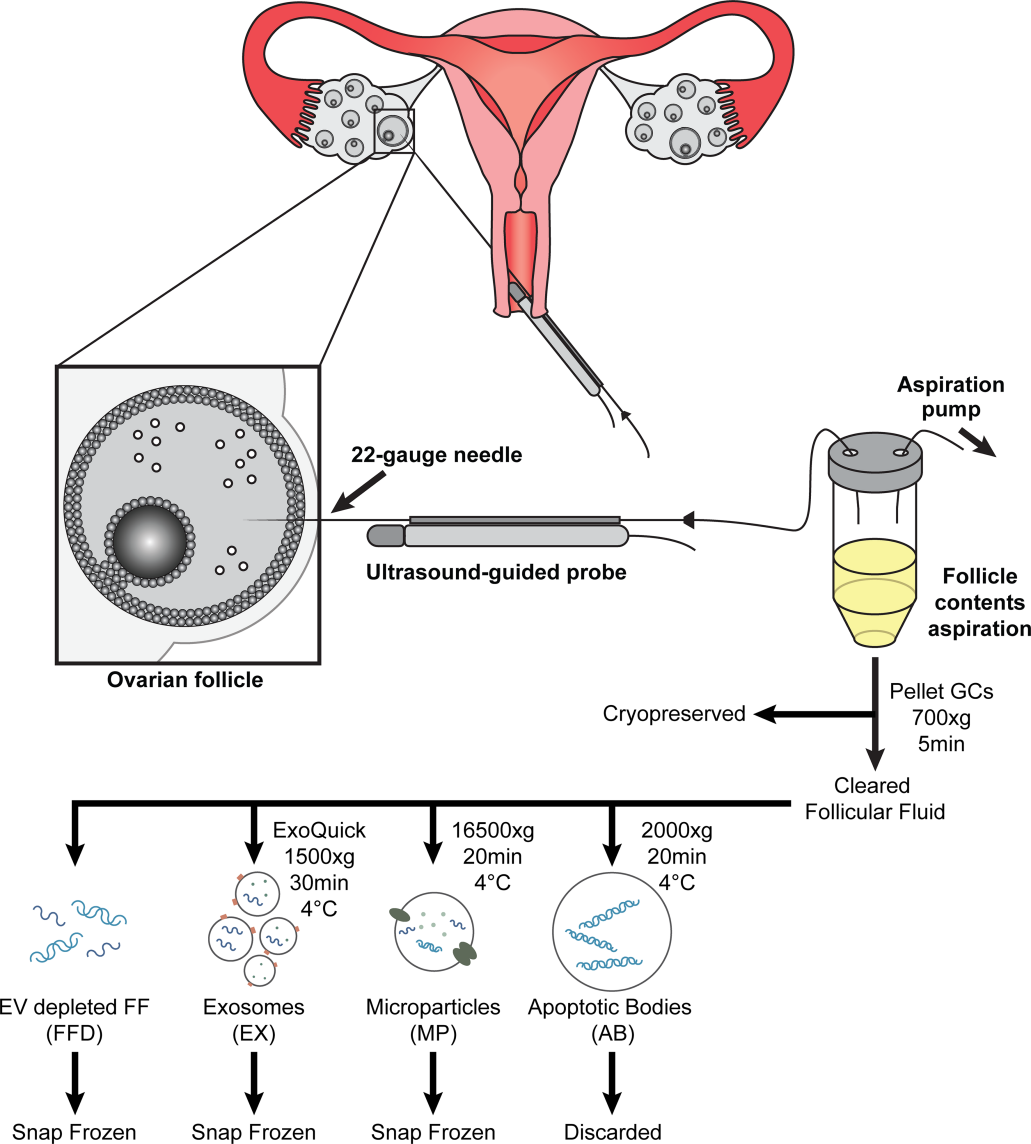
Diagram of ultrasound-guided oocyte retrieval for *in-vitro* fertilization treatment. Follicular contents are aspirated (follicular fluid (FF), oocyte (Oo), and granulosa cells (GC)) and collected following oocyte isolation. The GCs are precipitated and cryopreserved for future RNA extraction. Cleared follicular fluid is further processed to isolate extracellular vesicles using differential centrifugation and precipitation and cryopreserved for future RNA extraction.

Three types of EVs were isolated separately using a modified differential centrifugation protocol (32, 33), followed by exosome precipitation. First, 1 ml of follicular fluid was centrifuged at 700 x g for 5 min at 4°C to pellet any dead cells or cell debris; the pellet was discarded. The resulting supernatant was transferred to a new tube and centrifuged again at 2000 x g for 20 min at 4°C to pellet apoptotic bodies (ABs); the AB pellet was discarded. The supernatant was removed and centrifuged at 16500 x g for 20 min at 4°C to pellet microparticles (MPs); the MP pellet was resuspended in 200 ul PBS and snap frozen at -80°C for future use. The resulting supernatant was filtered using 0.2 um Whatman GD/X filters (Millipore Sigma, ON, Canada) and the appropriate volume of ExoQuick (System Biosciences, CA, USA) was added to the cleared supernatant and incubated overnight at 4°C without mixing. Following incubation, exosomes (EX) were pelleted by centrifugation at 1500 x g for 30 min at 4°C; the EX pellet was resuspended in 200 ul PBS and snap frozen at -80°C for future use. The resulting supernatant, deemed follicular fluid depleted of EVs (FFD), was collected and snap-frozen at -80°C for future use. The experimental workflow is illustrated in **Figure 1**.

### 2.5 Nanoparticle tracking analysis

EV subpopulations were analyzed using the NanoSight LM10, green laser (532nm) (Malvern Analytical) and Merlin F-033B ASG-camera (Allied Vision Technologies, Stadtroda, Germany) to determine particle size and concentration. All samples were diluted 1:100 in PBS prior to analysis. Five measurements of 30 s each were taken with consistent acquisition settings (gain=2.3, camera level = 14). Data analysis was performed using NTA 3.1 software (NanoSight, Malvern Analytical) with consistent analysis settings (gain=3, detection threshold=4). Median particle size, concentration, and size distribution were obtained for all EV subpopulations.

### 2.6 Granulosa cell culture and enrichment

Previously collected and cryopreserved single leading antral follicle GCs were thawed in a 37°C water bath. Contaminating cryoprotectant was washed away with prewarmed DMEM/F12 media (ThermoFisher). The cells were then seeded on an uncoated 10 cm culture dish and cultured in DMEM/F12 + 2.5% FBS for 30 min (37°C, 5% CO_2_, 21% O_2_) to allow for contaminating cells to attach. Following this, the supernatant containing unattached enriched GCs was collected and pelleted at 700 x g for 5 min at 37°C. The cell pellet was washed with PBS to remove media contamination, resuspended in 350ul of lysis Buffer RL (Norgen Biotek, ON, Canada), snap-frozen on dry ice, and then stored at -80°C until RNA extraction. Granulosa cell purity has been previously assessed for leukocyte and fibroblast contamination by flow cytometry and qPCR and no significant population of either cell type has been found following the differential plating protocol (data not shown).

### 2.7 RNA extraction and quantification

Total RNA from EVs was isolated using the Total Exosome RNA and Protein Isolation Kit (ThermoFisher), according to the manufacturer’s instructions. Briefly, EV samples were lysed and denatured using Phenol : Chloroform, RNA was precipitated and bound to the spin column, contaminants were washed away with 3 serial washes, and the purified RNA was eluted in 100 ul of elution solution. The final RNA extract was stored at -80°C until use. Total RNA from GCs was isolated using the Norgen Total RNA Kit (Norgen Biotek), according to the manufacturer’s instructions. Briefly, GCs were lysed by passing 10x through a 28G needle, RNA was precipitated and bound to the spin column, contaminants were washed away with 3 serial washes, and the purified RNA was eluted in 40ul of elution buffer. The final RNA extract was stored at -80°C until use.

### 2.8 Small RNA library preparation and next-generation sequencing

Small RNA libraries are prepared from 150 ng of total RNA using the Small RNA Library Prep Kit for Illumina (Norgen Biotek), according to the manufacturer’s instructions. Briefly, 3’ adapters were ligated to the RNA and excess adapters were removed using the included column-based cleanup. Following cleanup, 5’ adapters were ligated; the input RNA is now flanked by 3’ and 5’ adapters which were used to reverse transcribe the RNA. Following RT, unique indices were added through 14 rounds of PCR amplification. The final indexed PCR product was cleaned up using the included column-based cleanup. The eluate was run on a 6% TBE gel for size selection. The expected library size is ~140bp and the corresponding band was excised from the gel and columns purified. The final libraries were quantified using a Qubit DNA High Sensitivity kit (ThermoFisher) and the size was determined using a 2100 Bioanalyzer High Sensitivity DNA Kit (Agilent Technologies, CA, USA). Normalized libraries were pooled (48 samples per pool), denatured, diluted to 0.8 pmol/l, and loaded onto a High Output (75 cycle) flow cell (Illumina, CA, USA); followed by sequencing (1 × 75 bp) on a NextSeq 550 (Illumina).

### 2.9 Bioinformatics and statistical analysis

#### 2.9.1 Data processing

Data was analyzed as previously described (34). The FASTX-Toolkit (version 0.0.13) was used for adapter and short sequence (<15 nt) removal, and quality filtering (overall Phred score >30). Small RNA annotation was performed using Unitas (version 1.7) (35), which used seqmap (version 1.0.13) to align RNA to the following databases: tRNA database (release 18.11.2020), piRNA cluster database (release 18.11.2020), Ensembl (release 101), EnsemblGenomes (release 35), tRF and tRNA-leader sequence databases (release 09.04.2019), SILVA rRNA databases (release 132), and miRbase (release 22) (36–40). Sequences generating a single read were removed and count matrices were assembled with awk scripts. Raw sequencing files (fastq) can be found at https://www.ncbi.nlm.nih.gov/bioproject/971183 Accession # PRJNA971183

#### 2.9.2 Differential expression

A count matrix was imported into R (R Development Core Team 2013) and DESeq2 (version 3.1) (41) was used to determine differentially expressed small RNAs. Counts were collapsed by unique annotations, excluding sequence variants (eg. isomiRs). The design formula included the patient, BMI, diagnosis, and particle type. Data was explored using principal component analyses (PCA) and unsupervised hierarchical clustering analyses using Pheatmap (version 1.0.12) and the complete linkage method. Small RNAs were considered differentially expressed if the FDR adjusted p-value was below 0.05 (FDR<0.05) and the log2 fold change either greater than 2 or less than -2 (2<log2FC<-2).

#### 2.9.3 Target enrichment and functional analysis

Target enrichment and functional analysis were assessed for significant differentially expressed miRNAs, using MIENTURNET (42). Briefly, comparisons with at least 10 differentially expressed miRNAs were analyzed referencing the miRTarBase database (version 9.0) of miRNA interactions. Significant interactions were defined as having an FDR adjusted p-value < 0.05 and 2 minimum interactions. The targets of the top 10 significantly enriched miRNAs were assessed for their participation in cellular processes and functions using the KEGG database (release 99.1).

### 2.10 NGS validation by qPCR

Ten miRNAs were chosen for validation from the list of those which were differentially expressed. The choice of miRNAs was based on previous annotations deeming them as biologically significant and/or implicated in the pathway analysis. 10 ng of RNA was reverse transcribed using the TaqMan Advanced miRNA cDNA Synthesis Kit, according to the manufacturer’s instructions. Pre-designed and validated TaqMan Advanced miRNA Assays (ThermoFisher) were used for validation of NGS results with hsa-mir-92a-3p as the reference. All miRNAs were assayed in duplicate using TaqMan Fast Advanced Master Mix (ThermoFisher) (polymerase activation at 95 °C for 20s; 40 cycles of 1s denaturation at 95 °C and 20s annealing/extension at 60 °C). Relative fold change (ΔΔCt) was employed to quantify gene expression (43). Data analysis was performed using GraphPad Prism (version 5.02). The list of assays used for validation are in **Supplemental Table S1**.

## 3 Results

### 3.1 Patient and sample characteristics

A total of 35 FF samples were collected from individual mature follicles from 35 individual patients with a mean age of 35.7 ± 0.7 years old, mean BMI of 26.7 ± 1.1 kg/m^2^, and the following mean hormonal levels [Anti-Mullerian Hormone (AMH), 43.8 ± 4.0 pmol/L; luteinizing hormone (LH) on trigger, 2.8 ± 0.4 mIU/mL; and estradiol (E2) on trigger,11576.0 ± 970.2 pmol/L]. All clinical parameters were matched across the 4 groups, except for AMH and BMI, which were statistically significantly different between their corresponding comparison groups. Patient demographics broken down into the 4 groups are presented in **Table S2**. All samples had sufficient sequencing reads, high average quality scores, and high sequence alignment rates sufficient for differential expression analysis, as per guidelines previously published for quality control of RNAseq experiments (44).

### 3.2 Follicular fluid snRNAs cluster by adiposity and PCOS diagnosis

**Figure 2:**
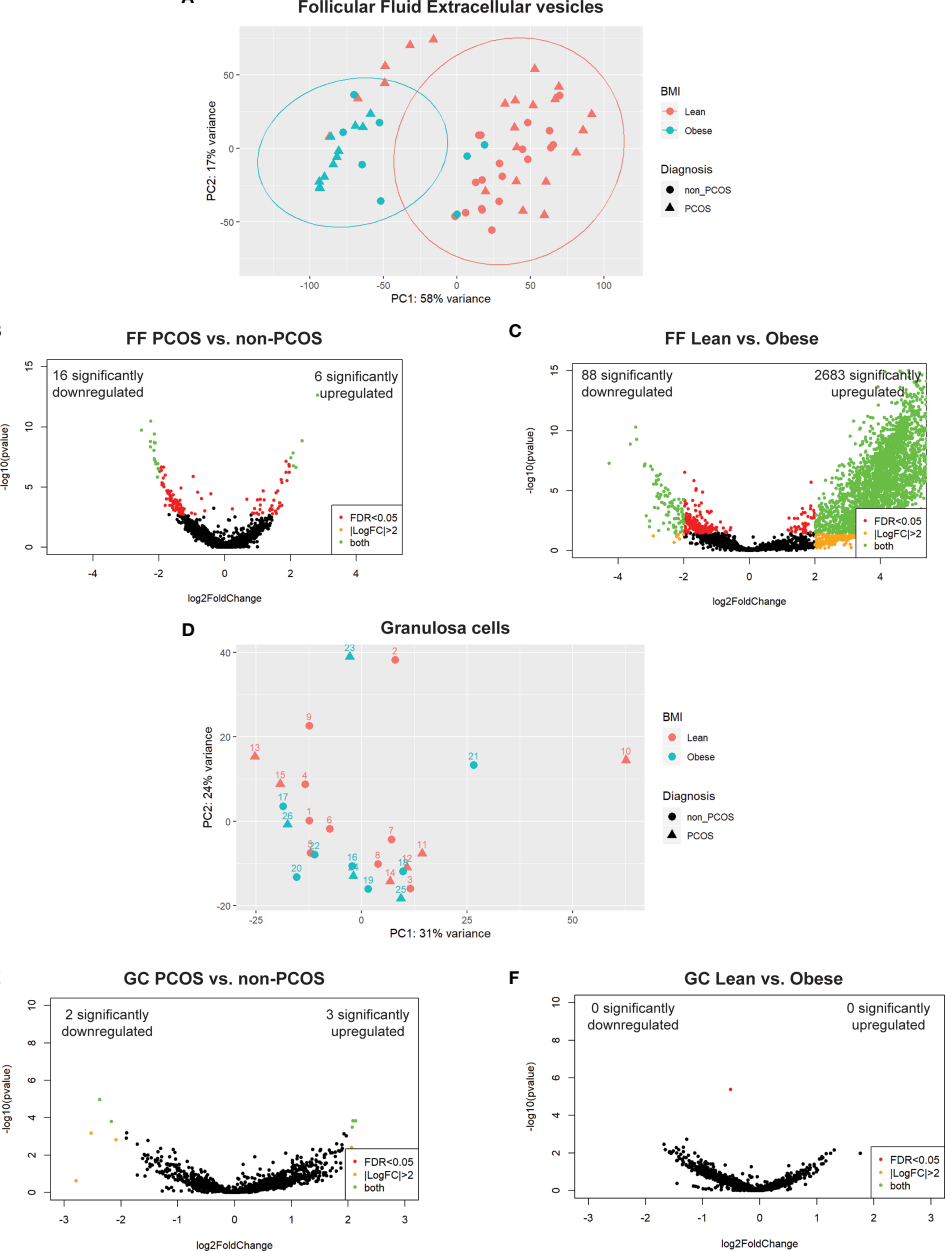
Principal component analysis (PCA) and differential expression. **(A)** PCA of all follicular fluid libraries shows significant separation along PC1 by BMI, depicted by colour, and no apparent effect of PCOS diagnosis, depicted by the shape. **(B)** DE analysis between PCOS and non-PCOS, regardless of particle type, using DESeq2; 22 snRNAs were differentially expressed (6 significantly upregulated (log2FC > 2 and FDR < 0.05), and 16 significantly downregulated). **(C)** DE analysis between Lean and Obese, regardless of particle type, using DESeq2; 2771 snRNAs were differentially expressed (2683 significantly upregulated, and 88 significantly downregulated). **(D)** PCA of all granulosa cell libraries does not show significant clustering by either BMI, depicted by colour, or PCOS diagnosis, depicted by the shape. **(E)** DE analysis between PCOS and non-PCOS using DESeq2; 5 snRNAs were differentially expressed (3 significantly upregulated, and 2 significantly downregulated). **(F)** DE analysis between Lean and Obese using DESeq2 identified no differentially expressed snRNAs. Differentially expressed snRNAs are depicted by the green dots in all volcano plots **(B, C, E, F)**.

A total of 4674 unique snRNAs were detected in FF regardless of group. PCA on all samples, prior to stratifying by extracellular vesicle type, showed clear clustering along PC1 associated with the participant’s BMI (**Figure 2A**). PC1 accounted for 58% of the variability observed in the dataset, indicating that BMI has the largest effect on FF profiles. Following this, differential expression analysis was conducted between all PCOS and non-PCOS samples. This analysis identified 6 significantly upregulated and 16 downregulated snRNAs (**Figure 2B**). When comparing all lean and obese samples, we identified 2683 significantly upregulated and 88 downregulated snRNAs, further illustrating the significant effect adiposity has on FF snRNA profiles (**Figure 2C**).

### 3.3 Granulosa cell snRNA profiles do not reflect differences in adiposity or PCOS diagnosis

A total of 2875 unique snRNAs were detected in GCs regardless of group. PCA on all samples showed no clustering based on either adiposity or PCOS diagnosis (**Figure 2D**), indicating that GC miRNA expression is not affected by BMI or PCOS diagnosis. Differential expression analysis between PCOS and non-PCOS GC samples identified 3 significantly upregulated and 2 downregulated snRNAs (**Figure 2E**). There were no significant differentially expressed snRNAs when comparing Lean vs Obese GC samples (**Figure 2F**).

### 3.4 miRNA is the most abundant biotype in extracellular vesicles

**Figure 3:**
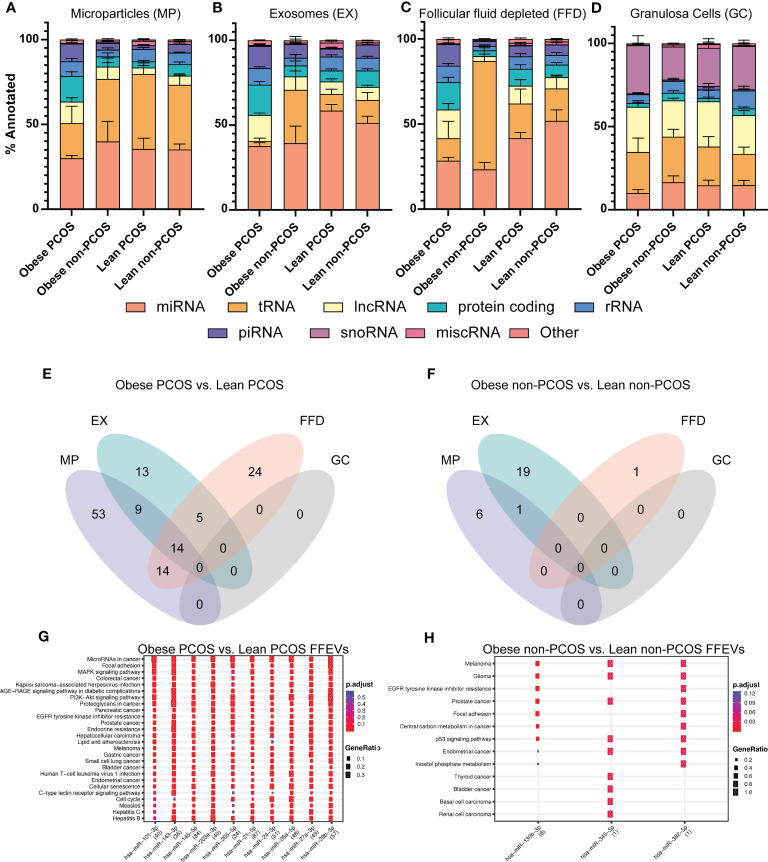
snRNA biotype distributions and differentially expressed snRNAs in subgroups. The proportion of annotated reads mapping to the indicated snRNA biotypes across the four subgroups **(A)** Microparticles (MP), **(B)** Exosomes (EX), **(C)** Follicular fluid depleted (FFD), and **(D)** Granulosa cells (GC). Venn diagram of differentially expressed miRNAs identified in each particle/cell type across two comparisons **(E)** Obese PCOS vs Lean PCOS, **(F)** Obese non-PCOS vs Lean non-PCOS. The colours correspond to the particle/cell type the miRNA was differentially expressed in. Overlapping regions indicate miRNAs that were differentially expressed in more than one particle/cell type. Functional analysis was conducted on the top 10 enriched targets and the associated pathways were determined using the KEGG database in **(G)** Obese PCOS vs. Lean PCOS and **(H)** Obese non-PCOS vs. Lean non-PCOS. The colour of the dot represents the adjusted p-value (FDR), whereas the size of the dots represents gene ratio (number of annotated targets in each KEGG annotation over the total number of recognized targets).

We next analyzed the overall snRNA biotype distribution captured from the three extracellular vesicle types and granulosa cells to determine the most prevalent classes as well as to identify selective packaging of specific biotypes in each EV type (**Figures 3A-D**). In the microparticle, exosomes, and follicular fluid depleted fractions, miRNA was the most abundant biotype, regardless of adiposity or PCOS diagnosis, with a mean of 35.2% ± 3.3%, 46.7% ± 3.0%, and 36.3% ± 3.3%, respectively; this was followed by tRNAs, protein-coding fragments, and long non-coding fragments (lncRNAs). All together these biotypes make up an average 83.3% of snRNAs identified. Interestingly, there was an observed enrichment in miRNAs in the exosomes, regardless of adiposity or PCOS diagnosis. In granulosa cells, the most abundant biotype, regardless of adiposity or PCOS diagnosis (mean 24.9% ± 2.0%), was lncRNAs followed closely by both snoRNAs and tRNAs, and then miRNA, which together make up 86.8% of snRNAs identified.

### 3.5 Adiposity significantly alters the miRNA signature in all extracellular vesicles but not in GCs

A total of 132 miRNAs were differentially expressed between obese PCOS vs lean PCOS groups, regardless of vesicle type. Fifty-three, 13, and 24 miRNAs were uniquely expressed in the MP, EX, and FFD fractions, respectively. Fourteen were differentially expressed in all fractions (**Figure 3E**). There were no differentially expressed miRNAs in the GCs. In the obese non-PCOS vs. lean non-PCOS comparison, 6, 19, and 1 miRNA were uniquely expressed in the MP, EX, and FFD fractions, respectively. One was differentially expressed in both the MP and EX fractions. There were no common DE miRNAs across all fractions and no DE miRNAs in the GCs (**Figure 3F**).

The comparisons of obese PCOS vs. obese non-PCOS and lean PCOS vs. lean non-PCOS are found in **Figure S1**. The top differentially expressed miRNAs in the obese PCOS vs. lean PCOS and obese non-PCOS vs lean non-PCOS comparisons are presented in **Tables S3**, **S4**, respectively. To determine the functional significance of these miRNAs, we performed target prediction, enrichment and functional analysis using all differentially expressed miRNAs. The pathways that are targeted by the selectively packaged and released miRNAs in obese PCOS vs. lean PCOS include genes involved in cell survival and apoptosis, leukocyte differentiation and migration, and several canonical signalling pathways, including JAK/STAT and MAPK signalling (**Figure 3G**). The pathways that are targeted by the selectively packaged and released miRNAs in obese non-PCOS vs. lean non-PCOS include genes involved in cell survival and apoptosis, p53 signalling, and various cancers (**Figure 3H**). Gene targets are included in **Table S5**.

### 3.6 miRNAs significantly enriched in obese PCOS EVs target genes involved in cell signalling and apoptosis

**Figure 4:**
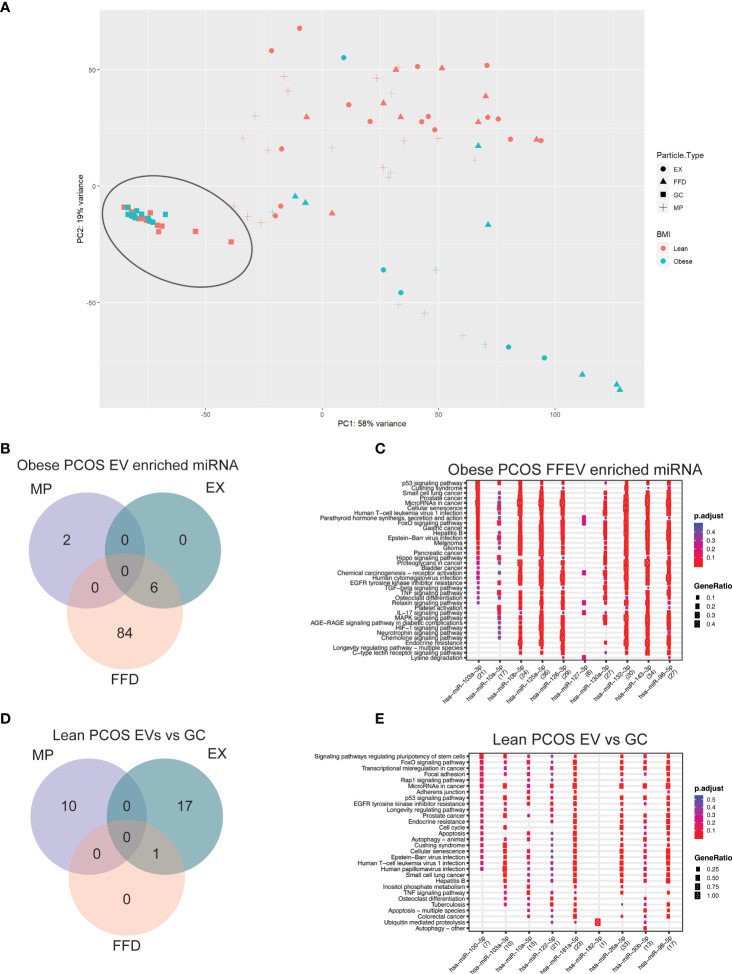
Principal component analysis (PCA) and differential expression in Granulosa cells. **(A)** PCA of granulosa cell and follicular fluid libraries shows significant separation along PC1 by follicular fluid extracellular vesicle or granulosa cell, depicted by the shape. The previously observed clustering based on adiposity is reproduced here in the follicular fluid samples and shows significant separation along PC2. **(B)** Venn diagram of miRNAs differentially expressed in the obese PCOS group significantly enriched in EVs compared to the corresponding granulosa cells. The colours correspond to the extracellular vesicle type the miRNA was detected in. Overlapping regions indicate miRNAs that were detected in more than one extracellular vesicle type. **(C)** Functional analysis was conducted on the top 10 enriched targets and the associated pathways were determined using the KEGG database in Obese-PCOS EVs. The colour of the dot represents the adjusted p-value (FDR), whereas the size of the dots represents gene ratio (number of annotated targets in each KEGG annotation over the total number of recognized targets). **(D)** Venn diagram of miRNAs differentially expressed in the lean PCOS group significantly enriched in EVs compared to the corresponding granulosa cells. **(E)** Functional analysis was conducted on the top 10 enriched targets and the associated pathways were determined using the KEGG database in Lean-PCOS EVs.

We next analyzed the differences between paired extracellular vesicle fractions and their corresponding granulosa cells to determine if there are specific miRNAs that are packaged and released into the FF. Using PCA, the GC samples are tightly clustered, regardless of diagnosis or adiposity, and separation between GC and EVs was observed along PC1, accounting for 58% of the variability in the dataset (**Figure 4A**). Furthermore, we once again observed separation based on adiposity in the EV samples along PC2, accounting for 19% of the variability in the dataset. Due to the large difference between GC and EV samples, we performed differential expression analysis to determine if there are specific miRNAs that are being either selectively packaged into EVs, or miRNAs being selectively retained in the GCs. In the obese PCOS EVs, we observed 2 miRNAs that were enriched in MP samples alone, 84 in FFE samples, and 6 common to both the EX and FFE samples (**Figure 4B**).

To determine the functional significance of these miRNAs, we performed target prediction, enrichment and functional analysis using differentially expressed miRNAs. The top 15 miRNAs, where applicable, were used for functional analysis (**Table S6**). The pathways that are targeted by the selectively packaged and released miRNAs in EVs include genes involved in p53 signalling, cell survival and apoptosis, several canonical signalling pathways, including FOXO, Hippo, TNF, and MAPK signalling (**Figure 4C**). A similar analysis on the miRNAs packaged in EVs from lean PCOS we observed 10 miRNAs that were enriched in MP samples alone, 17 in EX samples, and 1 common to both the EX and FFE samples (**Figure 4D**). When assessing the functional significance of these miRNAs, pathways involved in cell adhesion, pluripotency, and cell cycle were identified (**Figure 4E**). Gene targets are included in **Table S7**. Nine differentially expressed miRNAs detected using NGS were chosen for qPCR validation and showed similar direction and magnitude of expression for 8 the chosen targets (**Figure S2**).

## 4 Discussion

Our present study provides a novel method for the isolation from a single mature follicle of extracellular vesicles, followed by extraction, sequencing and analysis of snRNA from these-follicular fluid-derived extracellular vesicles (FFEVs). Using this methodology, we provide comprehensive profiling of snRNAs in FFEVs and GC of PCOS and non-PCOS patients, taking into account the potential independent effect of adiposity on these findings.

We observed that the vast majority of differences in the miRNA profile of the follicle are captured in the FF and not the GCs. This finding was further highlighted when assessing the biotype distribution of snRNAs in each FFEV type. We observed that miRNAs were the largest fraction of snRNA in all FFEV types across all but one group (obese non-PCOS - FFD). In contrast, miRNA was the fourth most abundant biotype in GC, behind lncRNA, snoRNA and tRNA species. Our results corroborate the findings of Goldie et al., 2014, who also reported that the proportion of miRNA in FFEVs were higher than that of the parent cell (45).

Focusing on miRNA differences in the obese vs lean groups, there was specific packaging of miRNA into unique FFEV types, with only 32% of the differentially expressed miRNAs being shared between more than one FFEV in the PCOS groups and no miRNAs shared between the FFEVs in the non-PCOS groups, indicating that there is selective packaging and release of miRNAs in a vesicle dependent manner. Selective packaging of miRNAs through the use of various RNA binding proteins (RBP) have been described in several diseases including chronic lung disease, diabetes mellitus, cancer, and heart disease (46). These RBPs bind to specific consensus sequences on miRNAs which facilitate selective shuttling into EVs (46). Based on our findings, both PCOS and obesity conditions appear to affect the miRNAs that are selectively packaged into specific classes of EVs across all comparisons assessed.

Throughout this study, adiposity has been highlighted as a major contributor to the differences observed between the snRNA profiles of these subjects, regardless of PCOS diagnosis, and by PCA accounts for 58% of the variability observed in the FFEV dataset. Adipose tissue is now recognized as an endocrine organ that impacts all facets of our biology and health (47). Not only do adipocytes secrete endocrine factors such as leptin or adiponectin, but it is also the largest source of circulating miRNAs, and adipose-derived miRNAs are considered a new class of adipokine (48). Adipose-derived miRNAs are highly stable due to their packaging in EVs, secondary structure, and by creating complexes with argonaute or high-density lipoproteins (HDL) (49–52). When comparing adiposity within PCOS FFEV samples, we were specifically interested in miRNAs upregulated in FFEVs of obese PCOS FF, as these miRNAs are more likely to be of adipose origin. To determine the biological effect of these miRNAs, we performed a functional analysis to identify the gene targets of these miRNAs and the biological pathways these genes are involved in, however no significant pathways were identified. Upon reviewing the literature, only one of the miRNAs enriched in FFEVs from obese PCOS patients has been previously characterized as adipose derived, miR-182, which is involved in brown adipocyte differentiation (53, 54). Interestingly, one of the highest predicted targets of miR-182 is *HAS2*, which regulates synthesis of hyaluronic acid. Hyaluronic acid is critical for cumulus cell expansion and is a well-documented marker of oocyte competence (55–58). Upregulation of this miRNA in obese FF may contribute to downregulation of *HAS2* observed in PCOS patients and may in part be contributing to the poor cumulus expansion observed in PCOS COCs (59). Of note, we identified 23 novel miRNAs which may be of adipocyte origin, however their cell of origin and functional significance in the ovary is currently unknown.

Next, we identified miRNAs that were specifically packaged and secreted into FF to discern the difference between individual FFEVs and matched GCs from the same follicle. Using PCA, we identified a tight cluster of all GC samples, which were distinct from the FFEV samples. In the obese PCOS group, we identified 92 miRNAs that were enriched in FFEVs compared to GCs, of which the majority were found unpackaged in FFDs. This finding was unique to the obese groups, with the lean groups having the majority of miRNAs packaged into either MP or EX, regardless of PCOS diagnosis. This indicates that there may be free-floating miRNAs in the follicular fluid, potentially protein bound, which are the cause or consequence of high adiposity in the patient. To determine the biological effect of these miRNAs, we performed target enrichment and functional analysis to identify the gene targets of these miRNAs and the biological pathways these genes are involved in. In the obese PCOS FFEVs, several well-characterized genes involved in apoptosis and cell survival were targeted by upregulated miRNA. Of note, three master regulators of gene expression, cell survival, and proliferation were identified as the top targets, *MYC*, *BCL2*, and *LIFR*, with 23, 15 and 14 miRNA interactions, respectively. MYC is a master regulator of gene transcription and is thought to regulate the expression of ~15% of all genes (60). Dysregulation of *MYC* expression is most associated with aggressive tumor growth and malignancy (60, 61). In PCOS, several critical pathways involved in cell growth, apoptosis, and signalling are dysregulated which include p52, p72/MYC, and MAPK (59). Of note, there were no significant pathways identified involved in glucose metabolism or insulin resistance.

BAX/BCL2-mediated apoptosis is a mechanism by which antral follicular growth arrests. In androgenized rats, the anti-apoptotic factor Bcl2 is poorly expressed in both antral and preantral GCs, with a correspondingly high level of BAX expression when compared to control rats, suggesting active apoptosis present in these cells (62). However, the expression of *BAX/BCL2* in human GCs has been shown to be higher in PCOS patients when compared to controls (7, 63). Interestingly, *BCL2* expression has been proposed to be regulated through the interaction between a circular RNA (circRNA) and a newly proposed miRNA involved in PCOS, miR-195-5p (63). However, in this current study, miR-195-5p was not identified as a differentially expressed miRNA in any of the comparisons.

The effect of altered expression of *LIFR* in the pathology of PCOS has not been previously characterized. However, in this study we identified several differentially expressed miRNAs that target *LIFR*, possibly implicating *LIFR* in the GC apoptosis observed in PCOS. *LIF/LIFR* has been implicated in promoting oocyte maturation, competence and increasing blastocyst yields in several animal models when added as an *in vitro* maturation supplement (64–67). In our study, *LIF* and *LIFR* were targeted by 4 and 14 miRNAs, respectively. This significant packaging and release of miRNAs in FFEVs targeting *LIF*/*LIFR* alters the FF composition and may be trafficked to the oocyte, resulting in altered oocyte maturation.

Our results imply that the selective packaging and release of these miRNAs that specifically target anti-apoptotic genes may be an attempt by the follicle to reduce GCs from undergoing the apoptosis and stave off premature follicle growth arrest observed in PCOS (68–70). By releasing the miRNAs into the FF, the negative inhibition of these miRNAs on the expression of these anti-apoptotic factors is released, allowing the cell to potentially reduce its apoptotic burden. Functional studies on how the miRNAs regulate this process may provide more information into the mechanism of follicle atresia in PCOS.

In conclusion, using a novel sequencing method, this study is the first to profile the small RNA in EVs from a single follicle. In addition, we successfully profiled with high fidelity all classes of snRNAs from MP, EX, FFD, and GCs from matched PCOS and non-PCOS patients. We also highlighted the significant impact adiposity has on snRNA profiles of PCOS and non-PCOS patients, with adiposity driving the observed snRNA differences. Finally, we identified miRNAs that are specifically packaged into EVs and secreted into the FF. We also propose a potential mechanism by which GCs selectively package and release miRNAs targeting anti-apoptotic genes in PCOS, consequently attenuating GC apoptosis and early onset follicular demise. This study has demonstrated that when investigating the pathophysiology of PCOS, it is critical to take into account the potential impact of adiposity on the ovary. This study is limited by its sample size, in part due to the inclusion of only PCOS patients who were not prescribed metformin. To overcome the limited sample size, we rigorously matched participants by all available confounding variables, thus minimizing inter-patient variability. Further, this study was not designed to determine the functional significance of the differentially expressed snRNAs however, it does provide a compelling evaluation of the impact obesity and PCOS has on signalling in the follicular niche. Last, this study was heavily focused on assessing the effect of obesity and PCOS on the expression of miRNAs however, this study contributes a large and biologically relevant dataset to the field that can be mined in future to determine the impact of other snRNAs on PCOS. As bioinformatic tools advance and with increased knowledge of these snRNAs increase, we predict that the importance of this dataset will become increasingly evident.

## Data availability statement

Publicly available datasets were analyzed in this study. This data can be found here: http://www.ncbi.nlm.nih.gov/bioproject/971183.

## Ethics statement

The studies involving human participants were reviewed and approved by University of Toronto Research Ethics Board Approval # 29236 and 29237. The patients/participants provided their written informed consent to participate in this study.

## Author contributions

BW and RS designed this study. BW and MS performed the experiments. SR performed the bioinformatic analyses, with input from BW. BW took the lead on interpreting the results, with support from RS, SR, BT, and CL. SJ collected, processed, and released samples and de-identified clinical data. BW wrote the manuscript with input from all co-authors. All authors contributed to the article and approved the submitted version.

## Supplementary material

Supplementary Figure 1 Venn diagram of differentially expressed miRNAs identified in the remaining two comparisons **(A)** Obese-PCOS vs Obese non-PCOS, **(B)** Lean PCOS vs Lean non-PCOS. The colours correspond to the particle/cell type the miRNA was differentially expressed in. Overlapping regions indicate miRNAs that were differentially expressed in more than one particle/cell type. **(C, D)** Target enrichment and functional analysis of miRNAs enriched in extracellular vesicles when compared to GC. Target enrichment using the miRTarBase database of target interactions in **(C)** Obese-PCOS EVs and **(D)** Lean PCOS EVs. The colours represent the adjusted p-value (FDR) of the target enrichment. A target gene is considered significant if the enrichment FDR<0.05 and the number of interactions > 2. Functional analysis was conducted on the top 10 enriched targets (where possible).

Supplementary Figure 2 qPCR validation of NGS results. Nine miRNA targets (and one reference target; hsa-mir-92a-3p) were chosen for validation from the list of differentially expressed targets. The choice of targets was based on previous annotations deeming these targets as biologically significant and/or identified by pathway analysis. All targets were assayed in duplicate and relative fold change (ΔΔCt) was employed to quantify gene expression. The list of primers and probes used for validation are given in **Supplemental Table S1**.
